# Affect systems, changes in body mass index, disordered eating and stress: an 18-month longitudinal study in women

**DOI:** 10.1080/21642850.2017.1316667

**Published:** 2017-04-18

**Authors:** N. Kupeli, S. Norton, J. Chilcot, I. C. Campbell, U. H. Schmidt, N. A. Troop

**Affiliations:** ^a^ Marie Curie Palliative Care Research Department, University College London, London, UK; ^b^ Health Psychology Section, Institute of Psychiatry, Psychology and Neuroscience, King’s College London, London, UK; ^c^ Section of Eating Disorders, Institute of Psychiatry, Psychology and Neuroscience, King’s College London, London, UK; ^d^ Department of Psychology, University of Hertfordshire, Hatfield, UK

**Keywords:** Stress, weight, disordered eating, affect regulation, longitudinal

## Abstract

**Background:** Evidence suggests that stress plays a role in changes in body weight and disordered eating. The present study examined the effect of mood, affect systems (attachment and social rank) and affect regulatory processes (self-criticism, self-reassurance) on the stress process and how this impacts on changes in weight and disordered eating.

**Methods:** A large sample of women participated in a community-based prospective, longitudinal online study in which measures of body mass index (BMI), disordered eating, perceived stress, attachment, social rank, mood and self-criticism/reassurance were measured at 6-monthly intervals over an 18-month period.

**Results:** Latent Growth Curve Modelling showed that BMI increased over 18 months while stress and disordered eating decreased and that these changes were predicted by high baseline levels of these constructs. Independently of this, however, increases in stress predicted a reduction in BMI which was, itself, predicted by baseline levels of self-hatred and unfavourable social comparison.

**Conclusions:** This study adds support to the evidence that stress is important in weight change. In addition, this is the first study to show in a longitudinal design, that social rank and self-criticism (as opposed to self-reassurance) at times of difficulty predict increases in stress and, thus, suggests a role for these constructs in weight regulation.

## Introduction

Bodyweight regulation is determined by a combination of genetic, physiological, environmental and psychological factors (Bessesen, 2011). It does not always follow a natural course and problematic weight regulation occurs in both eating disorders (EDs) and obesity. The prevalence of obesity and disordered eating (DE) are increasing (Craig & Hirani, 2010; Hay, Mond, Buttner, & Darby, 2008). It is therefore important to understand the factors that may cause these changes. In this introduction we present evidence for the link between these changes and the occurrence of stress as well as affect regulatory systems that impact on the stress process. We then integrate this evidence in a longitudinal study using a large sample of community-based women.

DE includes overeating as well as unhealthy weight control practices such as skipping meals, fasting, purging and using appetite suppressants and laxatives. Overeating as a result of disruption to healthy weight control practices (e.g. in response to emotional, situational or environmental cues such as the availability of palatable food) is also related to obesity and weight gain over time (Hays et al., 2002). Unhealthy weight control strategies contribute to greater weight gain compared to the use of healthy weight control behaviours such as increasing physical activity (Savage & Birch, 2010).

Substantial fluctuations in weight, specifically weight gain, have been found to trigger binge eating and purging behaviours resulting in the onset of threshold or sub-threshold bulimia nervosa (Thomas, Butryn, Stice, & Lowe, 2011) and can have adverse health consequences (French et al., 1997). However, other studies have indicated that it is disturbed eating pathology that precedes weight change in women, with those who report DE behaviours being more likely to gain weight (Field et al., 2007).

Stress also relates to problematic weight regulation (for a review, see Wardle, Chida, Gibson, Whitaker, & Steptoe, 2011). Stress can influence weight through physiological changes (Dallman et al., 2003; Roberts, Troop, Connan, Treasure, & Campbell, 2007) as well as behavioural changes such as changes in diet (Roberts, Campbell, & Troop, 2014). Stress has also been associated with eating pathology (Ball & Lee, 2000; Bennett & Cooper, 1999) including disruption of dietary restraint resulting in overeating and weight gain (Wardle, Steptoe, Oliver, & Lipsey, 2000). However, stress is associated with the onset of anorexia as well as bulimia nervosa (Schmidt, Tiller, Blanchard, Andrews, & Treasure, 1997) and some researchers have found that participants eat less during periods of stress (Stone & Brownell, 1994). The relationship between stress and weight change is therefore a complex one. Nevertheless, since stress is a common factor that influences both body mass index (BMI) and DE, the present report focuses on those factors that are implicated in the stress process. Specifically, the role of affect systems of attachment and social rank, as well as affect regulation processes such as self-criticism/reassurance will be considered.

Secure attachments and social rank are key biosocial goals and are proposed to be evolved systems (Gilbert, 1995). Attachment refers to the bond that develops between an infant and its caregiver (Bowlby, 1977) and social rank refers to one’s perceived position in relation to others in a hierarchical structure (Gilbert, 1997). These systems are a means by which positive mood can be maintained (i.e. they regulate affect), for example by maintaining proximity with attachment figures at times of threat/stress (attachment) and regulating agonistic behaviours to reduce intra-group conflict (social rank). The development of secure attachments is proposed to help us to learn the skills needed to manage difficult emotions at times of stress, being able to self-soothe (or to reassure oneself) rather than be self-critical (a form of self-attacking, associated with shame and self-perceived low status; Gilbert, 2006). Self-criticism refers to one’s critical self-evaluations and has been related to stress-induced biological changes (Gruen, Silva, Ehrlich, Schweitzer, & Friedhoff, 1997) whilst self-reassurance refers to the ability to reassure oneself at times of failure and is related to better psychological health (Gilbert, Clarke, Hempel, Miles, & Irons, 2004). In addition, as the social rank system is based on social comparisons, developing the ability to self-reassure in response to negative self-evaluations can keep critical thoughts and feelings of the self at bay (Gilbert et al., 2004). Attachment insecurity resulting from inadequate care and adverse life events during childhood is related to problems with stress (Uchino, Cacioppo, & Kiecolt-Glaser, 1996). There is also evidence for EDs and DE being related to insecure attachment styles (for reviews see, O’shaughnessy & Dallos, 2009; Zachrisson & Skårderud, 2010) and self-perceived low social rank (e.g. Troop, Andrews, Hiskey & Treasure, 2014; Troop & Baker, 2008).

Although attachment, self-criticism/reassurance and social rank have been related to experiences of stress and reported to influence the development of eating pathology, these affect regulatory systems have not been examined in relation to changes in weight. Therefore, as the stress process has been found to contribute to weight change and eating pathology, it is important to assess how these affect regulatory systems and processes contribute to stress and its effect on weight and DE.

### Objectives

The primary objective, therefore is to examine the associations between changes in stress, BMI and DE. The secondary objective is to examine the psychological predictors of these changes.

## Method

### Design and participants

A longitudinal study consisting of four assessments over 18 months, each approximately 6 months apart was conducted. Participants were recruited from the general population using a variety of opportunistic sampling methods, including social networking sites (e.g. Facebook), newspapers and email circulars to students at two universities in London and the South of England. At baseline, participants were given the opportunity to take part in the study follow-up by providing their email address. A total of 1465 participants (308 males and 1157 females) were recruited at baseline. Since gender differences are present for weight change and DE behaviours over time, data for men and women were analysed separately. However, as fewer males took part in the study combined with a high attrition rate (numbers at the four time points were 308, 73, 45 and 44, respectively), the models tested experienced limited power and convergence problems and only female data are presented. Of 1157 females, 820 indicated that they would like to participate in the follow-up. Follow-ups took place within a mean of 2.9 months between time one and two, 6.4 months between time two and three and 6.7 months between time 3 and 4.

### Measures and procedure

All data were collected online using the Bristol Online Survey (BOS) facility and ethical approval was obtained from the relevant University ethics committee. Participants provided basic demographic information such as age, gender, height and weight measurements (to calculate BMI kg/m^2^), ethnicity, marital status, highest education qualification and occupation. The following scales were also completed:

The *Eating Disorder Examination Questionnaire* (EDE-Q; Fairburn & Beglin, 1994) is a 36-item questionnaire consisting of 22 items measuring global DE behaviours and 14 diagnostic items. For the purpose of the current study, only the 22 global eating behaviour items were used to give a total EDE-Q score with higher scores indicating more eating pathology. Items include ‘Have you tried to avoid eating food which you like in order to influence your shape or weight?’ and ‘Have you had a strong desire to lose weight?’ Internal reliability (*α*) for the EDE-Q in the present sample was .95.

The *Food Frequency Questionnaire* (FFQ; Cade & Margetts, 1988) is a measure of caloric intake and asks individuals to state how frequently they consume different food types rated on a 6-point Likert scale from ‘Rarely/Never’ to ‘2 or more times a day’. This scale was developed in a large U.K. community sample (Cade & Margetts, 1988). Scores on the FFQ were positively skewed and so were log transformed.

The *Forms of Self-Criticising/Attacking and Self-Reassuring Scale* (FSCRS; Gilbert et al., 2004) is a measure of self-reassurance (reassured-self [RS]) and two types of self-criticism, inadequate-self (IS) and hated-self (HS). This scale was validated in female students (Gilbert et al., 2004) and further validated in a community sample (Kupeli, Chilcot, Schmidt, Campbell, & Troop, 2013). Items include ‘I am easily disappointed with myself’ and ‘I am gentle and supportive with myself’. The present study used an 18-item version derived from a confirmatory factor analysis (Kupeli et al., 2013) (*α*’s for the RS, IS and HS scales were .89, .89 and .84, respectively).

The *Perceived Stress Scale-4* (PSS-4; Cohen & Williamson, 1988) is a 4-item measure assessing perceptions of stress over the last month. Participants rate each item on a 5-point scale indicating the degree to which they appraise situations as stressful over the last month. This scale was validated in a large U.S. community sample (Cohen & Williamson, 1988) and recently validated in the U.K. (Warttig, Forshaw, South, & White, 2013). Items include ‘In the last month, how often have you felt that you were unable to control the important things in your life?’ and ‘In the last month, how often have you felt that things were going your way?’ Positive items were reversed and high scores indicated more stress (*α* = .80).

The *Short Depression-Happiness Scale* (SDHS; Joseph, Linley, Harwood, Lewis, & McCollam, 2004) is a 6-item measure of mood in which participants rate how they have felt over the last 7 days. This scale has been developed and validated in several student samples (Joseph et al., 2004). Items include ‘I felt dissatisfied with my life’ and ‘I felt that life was meaningless’. Lower scores indicate greater depressed mood and higher scores indicate greater happiness (*α* = .80).

The *Social Comparison Rating Scale* (SCRS; Allan & Gilbert, 1995) is an 11-item measure of social rank in which participants rate how they judge themselves in comparison with others on perceived rank, attractiveness and how they fit in with others. This scale has been developed and validated in student and patient samples (Allan & Gilbert, 1995). Items include ‘I am easily disappointed with myself’ and ‘I am gentle and supportive with myself’ (*α* = .93).

The *Vulnerable Attachment Style Questionnaire* (VASQ; Bifulco, Mahon, Kwon, Moran, & Jacobs, 2003) is a measure of insecurity of attachment. Participants rate statements assessing behaviours, emotions and attitudes which concern the way they feel about themselves in relation to others. The questionnaire was developed and validated in a community sample of 242 women selected for psychosocial risk factors for depression (Bifulco et al., 2003) and further validated in a U.K. community sample (Kupeli et al., 2015). Items include ‘I rely on others to help me make decisions’ and ‘I find it hard to trust others’. Though originally a 22-item scale, the present study uses a 14-item version derived from a confirmatory factor analysis (Kupeli et al., 2015) (*α* = .77).

### Statistical analysis

Latent Growth Curve Models (LGCM) using Mplus version 6 (Muthén & Muthén, 2010) were conducted with analyses computed using Full Information Maximum Likelihood estimator (FIML). FIML assumes data are missing at random (MAR). Auxiliary variables were included in the model to strengthen the MAR assumption (Graham, 2009).

Prior to testing the primary and secondary objectives using multivariate models, it was necessary to conduct both unconditional (i.e. without covariates) and conditional univariate models. Unconditional univariate LGCMs for BMI, EDE-Q and PSS-4 were conducted to assess the intercept and slope of each of the variables. Conditional univariate (i.e. including covariates) LGCMs were conducted to examine the effect of psychological variables and caloric intake variables on the intercept and slope of BMI, EDE-Q and PSS-4, separately. In order to test the primary and secondary objectives, conditional multivariate models with the observed variables for the four time points for each of these variables were used to compute latent variables to represent the initial level and the changes in BMI, EDE-Q and PSS-4. The variables that were used as predictors for the conditional models were age, SDHS, VASQ, SCRS, IS, RS, HS and caloric intake as measured at baseline.

To assess how well the proposed model fits the sample data, Chi^2^ and several fit indices were examined. The Comparative Fit Index (CFI) and the Tucker Lewis Index (TLI) have values ranging between 0 and 1 with those >.95 indicating a reasonable fit (Hu & Bentler, 1999). The Root Mean Square Error of the Approximation (RMSEA) is another fit index which takes into account the error of approximation in the population and values ≤.06 indicate a good model fit (Hu & Bentler, 1999)

## Results

### Sample characteristics and drop-out

**Figure 1.**
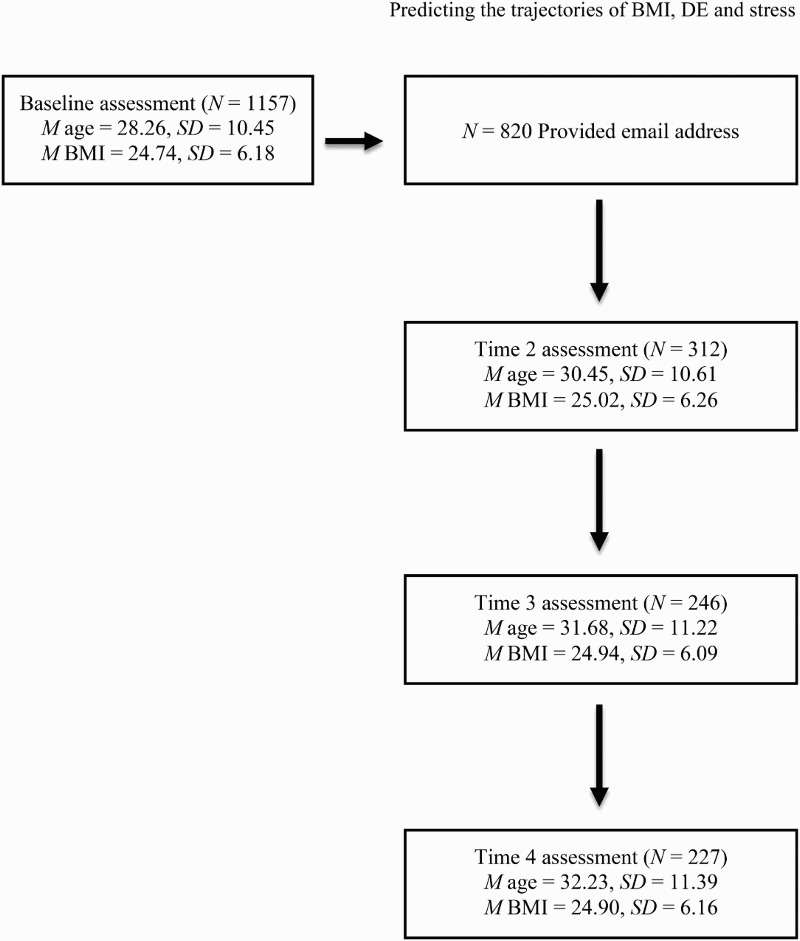
Flow diagram presenting the number of participants recruited at each time point and the corresponding mean age and BMI.

Sample characteristics are presented in Table 1, with study attrition information and mean age and BMI at each stage shown in Figure 1. Women who completed T2 were older (*t*(1155) = −3.67, *p* < .001) and with less attachment security (*t*(979) = 3.69, *p* <.001) compared to those who dropped out. These two variables were included in all of the models as a predictor or auxiliary variable to reduce the risk of bias.

**Table 1.** Baseline demographic variables (*N* = 1157).

Variable	% (*n*)
Ethnicity	
British	63.7 (737)
European	11.5 (133)
Indian	4.0 (46)
Bangladeshi	.6 (7)
Pakistani	2.4 (28)
Caribbean	1.6 (19)
African	2.9 (34)
Chinese	1.4 (16)
Mixed ethnicity	3.3 (39)
Other	8.5 (39)
Marital status % (*n*)	
Single	37.4 (433)
Married/Cohabiting	33.2 (385)
In a relationship	25.5 (295)
Divorced/Widowed	3.8 (44)
Highest education % (*n*)	
GCSE	7.0 (81)
A Levels	41.1 (476)
Bachelors	31.4 (363)
Postgraduate	19.2 (222)
None	1.3 (15)
Employment % (*n*)	
Employed	40.6 (469)
Studying	50.0 (579)
Unemployed	9.4 (109)

### Unconditional univariate LGCMs

Before testing the primary objective, unconditional univariate LGCMs were conducted to examine if there was a longitudinal change in the outcome variables and if baseline scores of these variables predicted a change (prior to identifying which factors predicted change in these variables).

BMI scores increased from a mean of 24.74 by .01 units per month (*p* = .01), indicating an increase in BMI of .18 over the 18 months of follow-up. The correlation between the intercept and slope was statistically significant (*r* = −.14, *p* = .03) indicating that the rate of change in BMI was dependent on the initial level of BMI, with those reporting a higher BMI at baseline more likely to experience a reduction in BMI over time. The variance of both the intercept (*b* = 37.79, *SD* = 6.15, *p* < .001) and slope (*b* = .01, *SD* = .1, *p* < .001) were statistically significant indicating that both baseline level and changes in BMI over time significantly varied across individuals in this sample. (*χ* ^2^ (6) = 5.34, *p* = .50, RMSEA <.001, CFI = 1.00, TLI = 1.00).

EDE-Q showed a mean score of 2.15 at baseline and the slope indicates that EDE-Q scores decreased by −.01 (*p* = .002) per month, indicating a reduction in EDE-Q scores of .18 over the 18 months of follow-up. The correlation between the intercept and slope was statistically significant (*r* = −.38, *p* < .001) indicating that the rate of change in EDE-Q scores was dependent on the initial level of EDE-Q and, as with BMI, those who reported higher EDE-Q scores at baseline were more likely to experience a reduction in their EDE-Q scores over time. The variance of both the intercept (*b* = 2.05, *SD* = 1.43, *p* < .001) and the slope (*b* = .001, *SD* = .03, *p* < .001) were statistically significant demonstrating that both baseline and changes in EDE-Q over time significantly vary across individuals in this sample. (*χ* ^2^ (5) = 10.34, *p* = .07, RMSEA = .03, CFI = .995, TLI = .994).

PSS-4 showed a mean score of 11.31 at baseline which decreased on average by .04 (*p* = .002), indicating a reduction in PSS-4 scores of .72 over the 18 months of follow-up. The correlation between the intercept and slope was not statistically significant (*r* = −.23, *p* = .49) indicating that the rate of change in PSS-4 scores is not dependent on the initial level of PSS-4. The variance of the intercept (*b* = 6.55, *SD* = 2.56, *p* < .001) was statistically significant indicating that baseline PSS-4 scores vary across individuals in this sample but the slope (*b* = .01, *SD* = .1, *p* = .25) was insignificant demonstrating that the change over time did not vary across individuals in this sample. (*χ* ^2^ (5) = 20.75, *p *< .001, RMSEA = .06, CFI = .919, TLI = .903).

### Conditional univariate LGCMs

Conditional univariate models were conducted to examine the effect of psychological and caloric intake variables on the intercept and slope of BMI, EDE-Q and PSS-4, separately. The conditional models were examined in light of the results of the unconditional models which showed that changes in BMI, EDE-Q and PSS-4 were significant in this sample with BMI increasing but EDE-Q and PSS-4 scores decreasing over time. However, as the main objective of the present study was to explore the effect of baseline and changes in BMI, EDE-Q and PSS-4 had on each other, the full results of the separate conditional univariate models will not be presented. Overall, the goodness-of-fit indices for the conditional models for BMI (*χ* ^2^ (26) = 25.36, *p* = .50, RMSEA < .001, CFI = 1.00, TLI = 1.00), EDE-Q (*χ* ^2^ (25) = 32.91, *p* = .13, RMSEA = .02, CFI = .995, TLI = .990) and PSS-4 (*χ* ^2^ (26) = 36.90, *p* = .06, RMSEA = .02, CFI = .988, TLI = .979) were acceptable.

### Multivariate LGCM

To test the primary and secondary objectives, a multivariate model examined the effect of psychological and caloric intake variables on the intercept (i.e. baseline levels) and slope of BMI, EDE-Q and PSS-4 and how the initial levels and changes in these three outcome variables influence each other. However, when running the parallel process model, BMI at T4 had little to no variance suggesting that there was no heterogeneity in this variable. Therefore, in order for the model to be interpreted the variance of this variable was fixed to zero.

**Figure 2.**
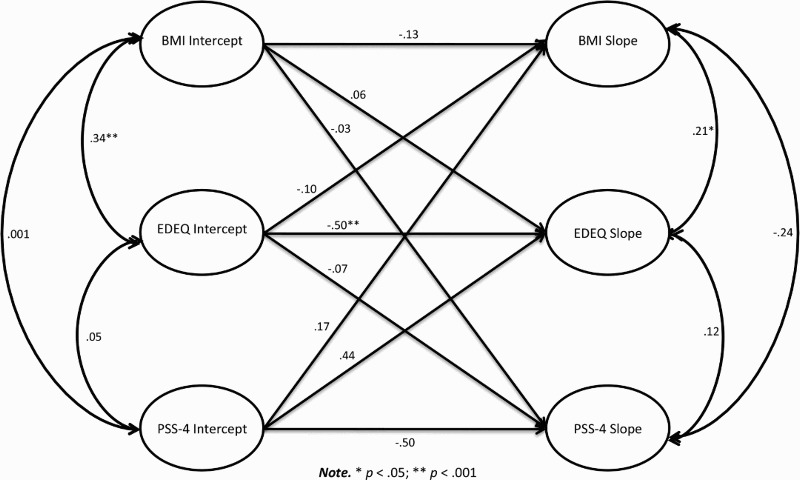
Path diagram of the multivariate model depicting the factor loadings and the correlations for the outcome latent variables.

The conditional multivariate model showed that baseline EDE-Q level was a significant negative predictor of the changes in EDE-Q scores (*β* = −.50, *p *< .001) indicating that higher levels of EDE-Q scores at baseline predicted a bigger decline in EDE-Q scores over time (Figure 2). A significant positive correlation between both initial levels of EDE-Q and BMI (*r* = .34, *p* < .001) and changes in EDE-Q and BMI (*r* = .21, *p* = .03) was found. This suggests that higher BMI at baseline was associated with higher initial EDE-Q scores and an increase in EDE-Q scores was associated with an increase in BMI. In addition to this, the model revealed a significant negative correlation between the changes in PSS-4 and BMI (*r* = −.24, *p* = .05) indicating that an increase in PSS-4 scores corresponded to a decrease in BMI over time. However, the correlation between the changes in EDE-Q and PSS-4 (*r* = .12, *p* = .39) was non-significant indicating that the changes in EDE-Q and PSS-4 are independent. The parameter estimates for the covariates in the conditional multivariate model are presented in Table 2, which was shown to have excellent goodness-of-fit properties (*χ* ^2^ (100) = 133.03, *p* = .02, RMSEA = .02, CFI = .993, TLI = .989).

**Table 2.** Parameter estimates and standard errors (standardized values) for the conditional multivariate model.

	BMI				EDE-Q				PSS-4			
	Intercept^a^		Slope^b^		Intercept^a^		Slope^b^		Intercept^a^		Slope^b^	
Variable	Est.	SE	Est.	SE	Est.	SE	Est.	SE	Est.	SE	Est.	SE
Age	.28**	.03	.04	.07	.07*	.03	.01	.08	−.08*	.03	.01	.10
VASQ	−.05	.04	−.11	.08	.05	.03	−.24*	.10	.16**	.03	.12	.13
SDHS	−.06	.05	.02	.16	−.18**	.04	.27	.21	−.58**	.05	.27	.39
SCRS	−.15*	.04	.05	.10	−.03	.04	−.16	.11	−.001	.04	−.34*	.14
FSCRS-IS	−.01	.05	.08	.10	.16**	.04	−.01	.12	.16**	.04	−.08	.16
FSCRS-RS	.07	.05	.06	.11	−.02	.04	−.03	.12	−.11*	.04	.03	.16
FSCRS-HS	.18**	.05	−.02	.10	.28**	.04	−.04	.12	−.004	.04	.30*	.15
FFQ	−.04	.03	.14^†^	.07	−.12**	.03	−.06	.08	−.003	.03	−.06	.09

Note: *FSCRS* = Forms of Self-Criticising/Attacking and Self-Reassuring Scale; *IS* = Inadequate-self; *RS* = Reassured-Self; *HS* = Hated-Self; *FFQ* = Food Frequency Questionnaire; *SDHS* = Short Depression-Happiness Scale; *SCRS* = Social Comparison Rating Scale; *VASQ* = Vulnerable Attachment Style Questionnaire.

^a^Initial level of outcome variable.

^b^Rate of change of outcome variable over time.

^†^ *p* = .05.

*p < .05.

**p < .001.

### Predictors of BMI

#### Cross-sectional at baseline

Age, SCRS and the HS component of the FSCRS were all significant predictors of the baseline level of BMI indicating that those who are older and report higher HS scores are more likely to report a higher BMI at baseline (see Table 2). However, those who report higher SCRS scores are more likely to report lower initial BMI.

#### Prospective

Caloric intake was marginally significant in predicting changes in BMI indicating that higher caloric intake at baseline is predictive of decreases in BMI over time (*p* = .05).

### Predictors of EDE-Q

#### Cross-sectional at baseline

Significant predictors of initial EDE-Q included age, SDHS, IS, HS and caloric intake. SDHS and caloric intake were found to have a negative effect and age, IS and HS had a positive effect on initial EDE-Q scores. These results suggest that those who report higher SDHS scores and caloric intake are more likely to have lower EDE-Q scores whereas those who are older and report higher IS and HS scores are more likely to report higher EDE-Q scores.

#### Prospective

VASQ was found to have a significant negative effect on the changes in EDE-Q. This suggests that higher scores on the VASQ predict a bigger decline in EDE-Q scores over time.

### Predictors of PSS

#### Cross-sectional at baseline

The conditional multivariate model demonstrated that the PSS-4 intercept was significantly predicted by age, VASQ, SDHS, IS and RS with age, SDHS and RS negative predictors and VASQ and IS positive predictors of initial PSS-4. This indicates that those who are older, report higher SDHS and RS scores are more likely to report lower PSS-4 scores but those who report higher VASQ and IS scores are more likely to report higher PSS-4 scores at baseline.

#### Prospective

Changes in PSS-4 scores was significantly predicted by SCRS and HS scores with higher SCRS at baseline predicting greater decreases in PSS-4 scores over time and higher HS scores at baseline predicting greater increases in PSS-4 scores over time.

## Discussion

The objectives of the present study were to examine the associations between changes in stress, BMI and DE and to determine the psychological predictors of these changes in a community sample of women over a period 18 months.

### Findings

Female participants experienced a small but significant increase in BMI and small but significant decreases in DE and stress over 18-months. Although the mean change in BMI is small (albeit significant), we were not interested in mean change at the sample level but heterogeneity in the changes at the individual level and the variance of the change over time, which was significant. The increase in BMI is consistent with previous reports of age-related weight gain (Bessesen, 2011), particularly in young women aged between 25 and 44 (Heitmann & Garby, 1999; Williamson, 1993) (the current sample had a mean age of 28). Previous research has also shown that, as women age, their disturbed eating behaviours decrease (Keel, Baxter, Heatherton, & Joiner, 2007). In our study, those with a higher BMI at baseline were more likely to experience a decrease in their BMI during the study while those who reported higher levels of DE and stress at baseline were more likely to experience a greater reduction in their DE and stress levels respectively over time.

High stress levels at baseline predicted a reduction in BMI over time. More importantly, however, an increase in stress levels over time was also associated with a reduction in BMI. Although a number of studies suggest stress generally induces overeating and weight gain in women (e.g. Roberts et al., 2007, 2014), in fact in a meta-analysis, the effect on weight gain was significant in men but not in women (Wardle et al., 2011). Our finding is, however, consistent with Stone and Brownell (1994) who found that participants eat less during periods of stress. However, causality is difficult to determine as these findings, though prospective, were correlational in nature.

Self-perceived lower social status and higher levels of self-criticism (specifically HS) at baseline predicted increases in stress levels over time. Consistent with previous findings, self-criticism predicted stress (Gruen et al., 1997) although in the present study this was specifically HS, not IS. However, the predictive value of self-perceived social status in relation to stress has not been looked at before although in a study on people exposed to trauma, a different aspect of social rank (social defeat rather than social comparison) did predict an increase in Posttraumatic Stress Disorder symptoms (Troop & Hiskey, 2013). Surprisingly, insecure attachment predicted decreases in DE over time which is in contrast to previous research showing that insecure attachment is related to greater DE and even the development and maintenance of EDs (O’shaughnessy & Dallos, 2009; Zachrisson & Skårderud, 2010). The difference here may be the measure and study design used; a prospective study in a community sample using a continuous measure of vulnerable attachment in adulthood versus retrospective interview studies in clinical samples in which attachment styles are inferred from the reporting of experiences in childhood (e.g. those using the Adult Attachment Interview: Main & Goldwyn, 1985). It is also possible that, in this community sample, insecure attachment motivated participants to initiate restorative processes that were not measured here and which resulted in improved (i.e. reduced) eating pathology. This is an issue for future research.

Some results were not as expected from the available literature. For example, low social status did not predict increases in DE (in contrast to Troop et al., 2014) and changes in stress did not predict changes in DE (e.g. in contrast to Ball & Lee, 2000; Bennett & Cooper, 1999; Schmidt et al., 1997; Wardle et al., 2000). However, these studies generally examined this association without considering covarying constructs. This demonstrates the advantage of considering changes in stress, weight and DE simultaneously in order to tease these factors apart in a multivariate analysis to identify genuine associations.

### Strengths and limitations

One limitation is that all measures were self-report. However, the high correlation (*r* = .95) between self-reported and objectively measured height and weight (Rowland, 1990) and the modest-to-high agreement between investigator-based and self-report versions of the Eating Disorder Examination (Pretorius, Waller, Gowers, & Schmidt, 2009; Wilfley, Schwartz, Spurrell, & Fairburn, 2000) mitigate this.

Although the study did recruit a male sample, it was not possible to analyse the data due to small sample size resulting in problems with model convergence. It was also not possible to combine the male and female data as there are many gender differences present in relation to BMI, DE and stress and men and women have been found to display different trajectories (Heatherton, Nichols, Mahamedi, & Keel, 1995; Keel et al., 2007; Williamson, 1993). Therefore, future studies should recruit a larger sample of men in order to examine if the same processes that influence BMI, eating pathology and stress in females occur for men.

The study recruited a non-clinical sample and the average age of 28 years old is older than those who typically go on to develop EDs (Currin, Schmidt, Treasure, & Jick, 2005). Therefore, our findings may not be generalizable to women with EDs. Finally, other factors that may have influenced weight change, DE and stress such as physical activity levels (Jakicic, 2011), smoking status (Freedman, Ron, Ballard-Barbash, Doody, & Linet, 2006), alcohol consumption (Rissanen, Heliövaara, Knekt, Reunanen, & Aromaa, 1991) and pregnancy and childbirth were not recorded.

A strength of the present study is its longitudinal nature which enabled the assessment of the changes that occurred over 18-months. Nevertheless, the present study may have missed the smaller fluctuations in BMI, DE and stress that may have occurred in between the 6-monthly follow-up points. However, research has suggested that weight change is relatively stable but, when changes in weight do occur, they tend to increase rather than decrease (Hopman et al., 2007). The present study is also the first to examine the related trajectories of BMI, DE and stress and the affect regulatory processes that influence these factors. Further, the recruitment of a large, community-based sample provided good statistical power. Nevertheless, the sample was predominantly white and young and thus may not be representative of the general population. Future research should aim to examine the processes driving BMI, eating pathology and stress in older adults and a more ethnically diverse sample.

### Implications

Although this study did not employ a clinical sample, the findings still have implications for practice. For example, weight loss programmes, eating disorder prevention programmes and stress management interventions should address the issues of perceived low social status, self-criticism and attachment. These may be either in terms of helping individuals to identify battles they can win or finding alternative contexts in which to achieve status (Sloman, 2008). Alternatively, interventions that render the effect of threats to social rank as less important may also be useful. For example, the ability to be self-soothing can tone down threat and self-criticism (Sloman, 2008). There is emerging evidence for the effectiveness of increasing self-compassion, of which the ability to self-soothe is a key component, including in EDs (Gale, Gilbert, Read, & Goss, 2014).

The present study focused on examining these processes amongst individuals who had varying bodyweights. However, studies suggest that prior weight loss is a strong predictor of subsequent weight gain (Colditz et al., 1990). In other words, since weight loss in overweight individuals is notoriously difficult to maintain, future research could usefully evaluate the role of the variables identified here in relation to weight loss maintenance.

## Conclusion

The current study is the first to integrate changes in stress, BMI and DE and simultaneously to identify affect regulatory predictors of these changes. In doing so, it furthers our knowledge in this area by showing that increases in stress are associated with decreases in BMI and that self-perceived low social status and HS predict increases in stress.
